# Impact of Hydrogen Bonding in Natural Cellulose Fibers on Plasmonic Nanoparticles

**DOI:** 10.3390/polym17233152

**Published:** 2025-11-27

**Authors:** Kunwara Techasuksakul, Prapakorn Khamphakun, Preeyanuch Srichola, Keowpetch Lobyaem, Chaiyaporn Sampoompuang, Thidarat Wangkham, Chanwit Kataphiniharn, Sorasak Phanphak

**Affiliations:** 1 Department of Industrial Physics and Medical Instrumentation, Faculty of Applied Science, King Mongkut’s University of Technology North Bangkok, Bangkok 10800, Thailand; s6204033920011@email.kmutnb.ac.th (K.T.); thidarat.w@sci.kmutnb.ac.th (T.W.); s6004033920012@email.kmutnb.ac.th (C.K.); 2 Kasetsart Agricultural and Agro-Industrial Product Improvement Institute, Kasetsart University, Chatuchak, Bangkok 10900, Thailand; prapakorn.kha@ku.th (P.K.); aappua@ku.ac.th (P.S.); aapttt@ku.ac.th (K.L.); aapcps@ku.ac.th (C.S.); 3 Cellulose for Future Materials and Technologies Special Research Unit, Department of Biotechnology, Faculty of Agro-Industry, Kasetsart University, Chatuchak, Bangkok 10900, Thailand; 4 Department of Physics, Faculty of Science, Kasetsart University, Chatuchak, Bangkok 10900, Thailand

**Keywords:** agave cellulose, plasmonic scattering, hydrogen bonding, dipole–dipole interaction

## Abstract

The aim of this study is to investigate the effect of hydrogen bonds in natural cellulose on luminescent properties, specifically focusing on controlling optical characteristics by tuning hydrogen bond interactions through the incorporation of light scattering from gold nanoparticles (AuNPs). The results from gold nanoparticles are that all samples exhibited strong plasmonic scattering within the 350–800 nm range, albeit with varying intensities. This study demonstrates that cellulose combined with AuNPs is highly effective for light scattering applications due to the reflective properties of cellulose and the surface plasmon resonance (SPR) effects of AuNPs. The enhancement of the autofluorescence signal increased about 2X relative to the Agave-AuNP sample and 67% higher than pure agave cellulose autofluorescence. Moreover, a quenching effect was observed in the mixture of cellulose, C_9_H_23_NO_3_Si, (APTES) and AuNPs attributed to hydrogen bond interactions, which diminished the light scattering properties. To understand the autofluorescence properties of cellulose and its interaction with metal nanoparticles, these composite materials are promising candidates for novel applications in bioimaging, sensing, and optoelectronic devices.

## 1. Introduction

Cellulose, the most abundant biopolymer on Earth, is composed of β-1,4-glycosidic-linked D-glucose units that form linear chains, which aggregate into microfibrils through extensive intra- and intermolecular hydrogen bonding. These hydrogen bonds are critical to the structural integrity, crystallinity, and unique mechanical properties of cellulose [1]. However, despite its remarkable physical properties, natural cellulose lacks intrinsic luminescent behavior, which limits its direct application in optical and photonic technologies [2]. Recent advances in materials science have explored various strategies to impart luminescent or light-modulating properties to cellulose, including chemical modification, doping with luminescent agents, and hybridization with nanomaterials [3]. Among these, the incorporation of metal nanoparticles, such as gold nanoparticles (AuNPs) have garnered significant attention due to their localized surface plasmon resonance (SPR), which can enhance light scattering and absorption within specific wavelengths. The plasmonic properties of AuNPs make them attractive candidates for sensors, optical coatings, and light-harvesting systems [4,5].

Metal-based nanoparticles (NPs), especially those composed of noble metals such as gold (Au), silver (Ag), and platinum (Pt), have attracted extensive interest due to their size-dependent optical, electronic, and catalytic properties. These nanoscale materials exhibit high surface-area-to-volume ratios, tunable surface chemistry, and unique quantum confinement effects, enabling distinctive interactions with electromagnetic radiation. Among their optical phenomena, localized surface plasmon resonance (LSPR) is particularly significant. LSPR arises from the collective oscillation of conduction electrons on the surface of nanoparticles in resonance with incident light, resulting in strong absorption and scattering at specific wavelengths. This resonance condition is highly sensitive to the size, shape, and composition of the nanoparticles, as well as the dielectric properties of the surrounding environment, enabling their use in a wide range of optical and sensing applications [6,7]. This localization facilitates significant field enhancements near the nanoparticle surface and underpins applications in biosensing, photothermal therapy, and fluorescence modulation [8].

In particular, gold nanoparticles (AuNPs) are widely used due to their biocompatibility and well-defined LSPR properties. A commonly employed synthesis method is the Turkevich method, which involves the reduction of chloroauric acid (HAuCl_4_) by trisodium citrate in a boiling aqueous solution, producing spherical AuNPs with sizes ranging from 10 to 20 nm [9]. Recent advancements in green methods, which use, utilizing plant extracts or biomolecules as reducing and stabilizing agents, provide environmentally friendly alternatives and facilitate functionalization for biological applications [10,11].

By integrating both cellulose and metallic nanoparticles into composite systems, the interaction between the host matrix and the embedded nanoparticles plays a pivotal role in determining the overall optical response. Cellulose, with its abundance of hydroxyl groups, offers a highly interactive matrix for the nanoparticles [12,13]. These hydroxyls groups remove can form hydrogen bonds, which can influence the spatial distribution of electrons on the nanoparticles surface, thereby affecting their optical properties. Consequently, controlling hydrogen bonding interactions within cellulose is a key factor in tuning the luminescence and light-scattering characteristics of cellulose-based nanocomposites [14,15,16].

Furthermore, the introduction of silane coupling agents, such as 3-aminopropyltriethoxysilane (APTES), facilitates additional chemical interactions that can either enhance or quench the optical performance of the composite. APTES is known to form hydrogen bonds with cellulose, potentially altering the microstructure and interfacial properties of the material [17,18]. However, such interactions may also introduce non-radiative pathways or interfere with SPR effects, leading to quenching phenomena [19]. In this context, APTES plays a dual role in hybrid nanomaterial systems. First, its amine (-NH_2_) group contributes to the improved dispersion and stabilization of gold nanoparticles, which is crucial for preserving their optical properties, particularly their localized surface plasmon resonance (LSPR) behavior [20,21]. Second, APTES can modify the interfacial interactions between cellulose and nanoparticles by altering the local dielectric environment. This interfacial tuning has been shown to influence key optical properties, including absorbance intensity, spectral shifts, and scattering behavior [22].

This study aims to investigate the role of hydrogen bonding in natural cellulose in modulating the luminescent and light-scattering properties when hybridized with AuNPs. Specifically, we explore how tuning hydrogen bond interactions either through physical blending or chemical modification with APTES can affect the optical behavior of cellulose-AuNPs composites. Our results reveal that all cellulose-AuNPs samples exhibit pronounced plasmonic scattering within the range of 350–800 nm. However, the presence of APTES introduces hydrogen bonding interactions that result in a quenching effect, diminishing the light-scattering efficiency. These findings suggest that cellulose-AuNPs composites hold significant potential for applications in photonic materials, with their optical performance tunable through hydrogen bonding dynamics.

## 2. Materials and Methods

### 2.1. Materials

Agave cellulose (Agv.) was sourced from the Plant Processing and Innovation Development Group in Yang Hak Subdistrict, Pak Tho District, Ratchaburi Province, Thailand. Milli-Q water with a resistivity of 18.2 MΩ·cm was used for all aqueous solutions. 3-Aminopropyltriethoxysilane (APTES, 99%) was sourced from Sigma-Aldrich (St. Louis, MO, USA). Sodium hydroxide (NaOH, 97%) and hydrogen peroxide (H_2_O_2_, 55%) were purchased from Kemaus Pty Ltd. (New South Wales, Australia). Sodium silicate (Na_2_SiO_3_) was purchased from AppliChem GmbH (Darmstadt, Germany). Gold nanoparticles (AuNPs) were provided by the Materials Laboratory, Department of Physics, Faculty of Science, Kasetsart University, courtesy of Asst. Prof. Dr. Weeraphat Pon-On.

### 2.2. Soda AQ Pulping Process Description

The soda-anthraquinone (AQ) pulping method, as described in [23], was employed in this study. Agave residue served as the raw material, with varying concentrations of sodium hydroxide (NaOH) applied at 40 wt% relative to the Agave residue. A small amount of anthraquinone (AQ), equivalent to 2 wt% of the Agave residue, was added as a pulping catalyst. The mixture was pulped in water at 85 °C for 4 h to produce a pulp slurry. The resulting Agave pulp was then filtered through a screener to remove large particles and impurities. Then, the Agave pulp was bleached using 20% hydrogen peroxide (H_2_O_2_) and 1% sodium silicate (Na_2_SiO_3_) at 85 °C for 2 h [24].

### 2.3. Silanization Process

**Figure 1:**
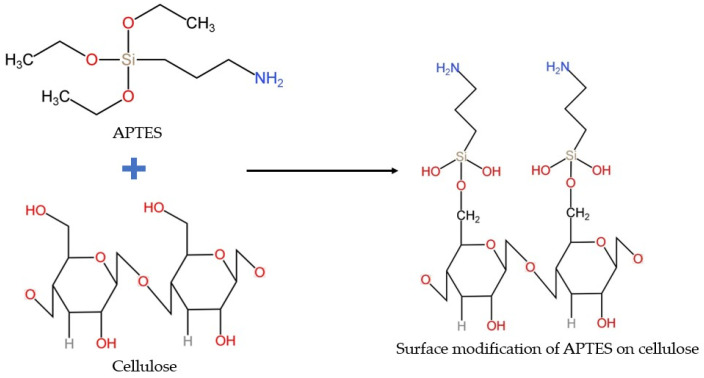
Silanization process for APTES–cellulose conjugation.

The silanization of cellulose was performed using 3-aminopropyltriethoxysilane (APTES) to introduce functional amino groups onto the cellulose surface. Initially, the agave cellulose was thoroughly washed with distilled, deionized water to remove any residual impurities. For the silanization process, the dried cellulose was dispersed in a 0.1 wt% aqueous solution of 10 mL under constant stirring. The dispersion was then applied onto a glass slide and dried at 60 °C. APTES (10% *v*/*v*) was added directly onto the agave cellulose and incubated for 30 min. After incubation, the modified cellulose was washed repeatedly with distilled water to remove any unreacted silane molecules. Finally, The APTES-modified cellulose was maintained under reflux conditions at 70 °C for 1.5 h to ensure effective silane coupling (Figure 1).

The composite sample was prepared by drop-casting gold nanoparticles (AuNPs) onto an APTES-modified cellulose surface, followed by drying at room temperature. Unbound AuNPs were removed by washing with distilled water. For comparison, the Agave cellulose-AuNPs sample was prepared using the same procedure but applied directly to the unmodified cellulose surface.

### 2.4. Photophysical Properties

The absorption and fluorescence intensities were measured using a SPARK Multimode Microplate Reader. The absorption intensity was measured with absorbance measurement mode: wavelength 300–900 nm at wavelength step size 2 nm. The fluorescence Intensity was measured with fluorescence intensity scan top reading mode, with an emission wavelength range of 400–700 at the emission wavelength step size of 3 nm. The transmission was measured with the measurement mode of the transmission spectrometer in mode, with a scanning interval of 1.0 nm, an integration time of 50 ms, and a measurement range of 325–900 nm.

### 2.5. Atomic Force Microscopy (AFM)

Atomic Force Microscopy (AFM) was employed to investigate the surface morphology and nanoscale topographical features of the composite samples. Measurements were carried out using tapping mode under ambient air conditions to prevent sample damage and preserve its native structure. AFM analysis provided detailed information on surface roughness, fiber alignment, and nanoparticle distribution. The resulting topographical images were processed using Gwyddion software (version 2.63) to quantify surface roughness and height profiles. These nanoscale features were correlated with the degree of hydrogen bonding within the cellulose structure and their impact on nanoparticle dispersion and plasmonic response.

### 2.6. FT-IR

The prepared agave cellulose treated with APTES and AuNPs was characterized as follows: the functional groups of agave cellulose, APTES and AuNPs: Fourier-transform infrared (FT-IR) spectroscopy (Nicolet IR200 FT-IR Infrared Spectrometer, Thermo Scientific, Waltham, MA, USA) was employed to measure infrared radiation absorbance.

### 2.7. Particle Charge Detector (PCD)

The particle charge of Agv., Agv. + AuNPs, Agv. + APTES + AuNPs and Agv. + 2APTES + AuNPs samples was determined using a Mütek™ PCD-05 Particle Charge Detector (BTG Instruments GmbH, Weßling, Germany). The instrument was operated in automatic titration mode using a polyelectrolyte of opposite charge as the titrant. All measurements were performed in triplicate, and the results are reported.

## 3. Results and Discussion

**Figure 2:**
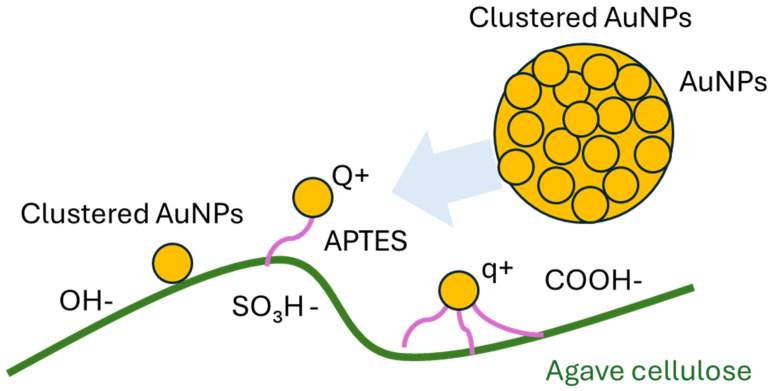
The schematic of clustered Au−nanoparticle localization on Agave cellulose.

The effects of H-bond on plasmonic nanoparticles were observed via photophysical investigation, where the morphological studies were exploited from the atomic force microscopy (AFM) and scanning electron microscope (SEM). To further investigate the presence of APTES and cellulose on AuNPs, FTIR spectroscopy was employed to verify the interactions between the nanoparticles and cellulose fibers. A schematic of clustered AuNPs on functionalized cellulose, illustrating different types of interactions, is shown in Figure 2.

### 3.1. Photophysical Properties from Absorption and Transmission Spectroscopy

**Figure 3:**
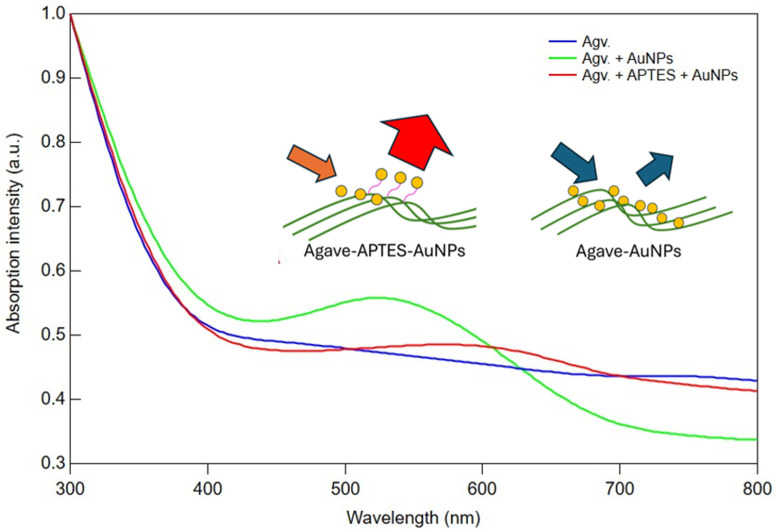
Absorption intensity of the samples under different conditions. The absorption of Agave shows different absorption due to the distance of AuNPs from the Agave cellulose. Cartoon insets reveal mechanisms how distance influence the plasmonic absorption.

The absorption spectra of Agv., Agv. + AuNPs, and Agv. + APTES + AuNPs were recorded over the range of 300–800 nm to investigate their optical properties and assess the impact of surface modifications and nanoparticle integration (Figure 3). All the samples exhibited an absorption spectrum in the UV region, characteristic of organic compounds or plant-based extracts. This is likely due to the presence of polyphenols, flavonoids, or other conjugated systems that absorb in the UV range. A distinct surface plasmon resonance (SPR) band of AuNPs was observed at around 530 nm for the Agv. + AuNPs sample, confirming the successful incorporation of gold nanoparticles into the Agave matrix. This change was further highlighted upon APTES modification, where the SPR peak shifted noticeably to approximately 600 nm, indicating nanoparticle aggregation and alterations in the local dielectric environment caused by the silane linker [25,26]. The observed red-shift was consistent with FDTD simulation results (Supplementary Figure S2). The absorption peak of AuNPs shifted from 530 nm to 600 nm when the AuNPs aggregated with APTES. The effect of APTES conjugation directly influences optical absorption.

**Figure 4:**
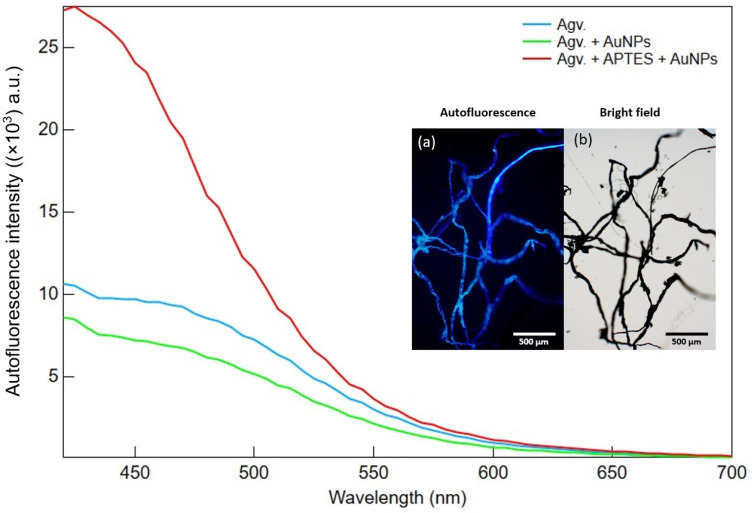
Autofluorescence intensity of the samples measured under different conditions, showing the variation in signal response. Inserts: (**a**) autofluorescence of untreated cellulose fibers and (**b**) corresponding bright−field image of the fibers for morphological reference.

In Figure 4, the fluorescence emission spectra of the Agave-based composites were displayed, together with autofluorescence and bright-field micrographs of cellulose fibers (Figure 4a,b). The Agv. sample exhibited a moderate emission band peaking between 400–460 nm, attributable to intrinsic autofluorescence from plant metabolites such as polyphenols and flavonoids [27]. When AuNPs were incorporated (Agv. + AuNPs), the autofluorescence intensity decreased relative to the cellulose matrix. This quenching is likely due to plasmon-induced non-radiative energy transfer processes (e.g., FRET and SET), whereby the excited dipole energy of fluorophores is dissipated into localized plasmons of AuNPs. The distance sensitivity of these processes suggests that fluorophore–AuNP proximity allows AuNPs to act as energy acceptors rather than enhancers [28,29].

To explain about the effect of cellulose-AuNPs interaction, the dipole-plasmonic energy transfer can be defined with the length constant $\left(L_{0}\right)$ [30] as

$$L_{0}={\left(\frac{A\varphi_{D}}{2\pi k^{2}}{\lambda_{f}}^{2}\right)}^{1/4},$$

where *A* is a unitless constant (0.525), $\varphi_{D}$ is the donor’s quantum efficiency function, k is the donor’s transition wave vector which is derived from donor’s transition frequency ω, and the $\lambda_{f}$ is metallic plasmonic wavelength. The efficiency varies with the factor of $1/(1+{\left(\frac{L}{L_{0}}\right)}^{4})$ from the dipole–dipole distance *L*. This reveals the relation of autofluorescence quenching or enhancement on the nanoparticles.

However, in the presence of APTES (Agv. + APTES + AuNPs), autofluorescence intensity increased dramatically, approximately threefold compared to both Avg. and AuNP-only samples. This enhancement implies that APTES facilitates better interfacial integration and more uniform nanoparticle anchoring. This behavior is further supported by fluorescence microscopy results (Supplementary Figure S1), where APTES-modified cellulose exhibited strong FITC labeling compared to unmodified fibers. In particular, the silane groups may form hydrogen bonds with hydroxyl and carbonyl moieties in the matrix, improving compatibility, while the amine-terminated surface supports a more homogeneous AuNP distribution. The more uniform dispersion helps prevent excessive proximity between fluorophores and AuNPs, thereby reducing quenching pathways and favoring plasmon-enhanced fluorescence (PEF) [31,32,33]. Overall, these findings indicate that while AuNPs alone tend to quench autofluorescence, functionalization with APTES supported by hydrogen bonding interactions can enhance autofluorescence and optical properties by improving nanoparticle distribution and optimizing the interface between the AuNPs and the Agv.-based matrix. As shown in Figure 4, the fluorescence intensity of the Agv. + APTES + AuNPs sample increased compared to the other conditions. The Agv. cellulose exhibited an autofluorescence intensity of approximately ~16,350 a.u., which decreased to ~8626 a.u. after AuNP incorporation due to quenching effects induced by the metallic nanoparticles. Surface functionalization with APTES led to a substantial enhancement of the fluorescence signal, reaching ~27,200 a.u. Notably, the emission maximum remained near 430–450 nm in all samples, indicating that the enhancement arises from interfacial and plasmonic coupling effects rather than from the introduction of new fluorophore species. Prior studies have shown that appropriately spaced plasmonic nanostructures embedded within biopolymer matrices can increase the radiative decay rate and amplify local electromagnetic fields, thereby enhancing autofluorescence yield [34]. Thus, our results strongly support a shift in photophysical balance from quenching to enhancement mediated by APTES-driven nanoparticle distribution and interfacial bonding.

**Figure 5:**
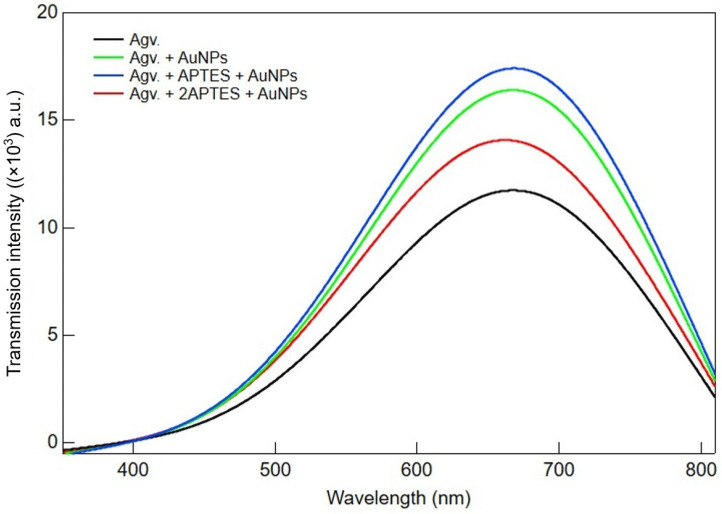
UV–Visible transmission spectra of Agave-derived cellulose (Agv.) and its modified forms: Agv. + AuNPs, Agv. + APTES + AuNPs, and Agv. + 2APTES + AuNPs.

The transmittance spectra of Agv., Agv. + AuNPs, Agv. + APTES + AuNPs and Agv. + 2APTES + AuNPs are presented in Figure 5. The Agv. sample exhibited the lowest transmittance intensity across the entire spectrum, with a maximum peak observed around 680–700 nm. Upon incorporation of AuNPs (Agv. + AuNPs), the transmittance intensity increased noticeably, indicating that the presence of gold nanoparticles enhanced light transmission due to improved material uniformity and reduced light scattering. Further functionalization with APTES (Agv. + APTES + AuNPs) resulted in a more pronounced increase in transmittance, suggesting that APTES not only stabilized the AuNPs but also improved their dispersion within the Agv. matrix. This uniform distribution may reduce both light absorption and scattering, thereby enhancing transmission. However, when the amount of APTES was doubled (Agv. + 2APTES + AuNPs), a slight decrease in transmittance was observed compared to the single-APTES sample. This suggests that excessive APTES may lead to nanoparticle clustering or increase the refractive index mismatch, thereby enhancing light scattering and slightly reducing transmission. Overall, these results indicate that an optimal number of APTES improves the dispersion of AuNP-containing Agv. composites, while excessive functionalization may adversely affect transmittance.

### 3.2. Morphological Studies Using Atomic Force Microscopy (AFM) and Scanning Electron Microscopy (SEM) Analysis

**Figure 6:**
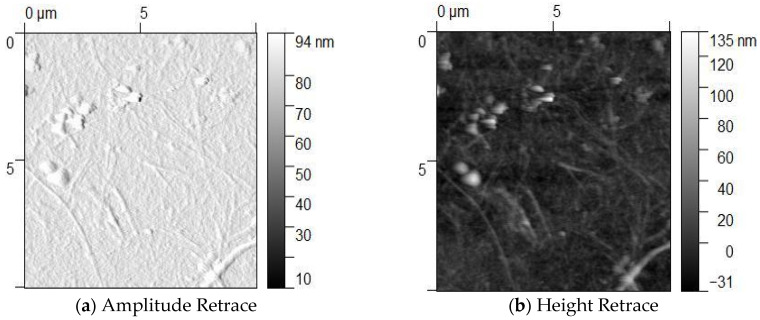
AFM image of Agv. with APTES and AuNPs indicate the retraction of AFM probe on (**a**) Amplitude and (**b**) Height.

The AFM analysis of Agv. modified with APTES and AuNPs revealed distinct surface characteristics (Figure 6). Amplitude values ranged from 30 to 94 nm, indicating a significant difference between the minimum and maximum measurements. This variation reflects a heterogeneous distribution of surface components and suggests relatively high surface roughness in the samples. The pronounced differences observed in amplitude further imply that the AuNPs are unevenly distributed and tend to form localized aggregates. In some regions of the height retrace images, height values exceed 100 nm, likely due to the accumulation or clustering of AuNPs on the cellulose surface. These results confirm the successful modification of Agave cellulose with AuNPs. However, the uneven distribution of nanoparticles across the surface may affect the material’s functional properties in future applications.

**Figure 7:**
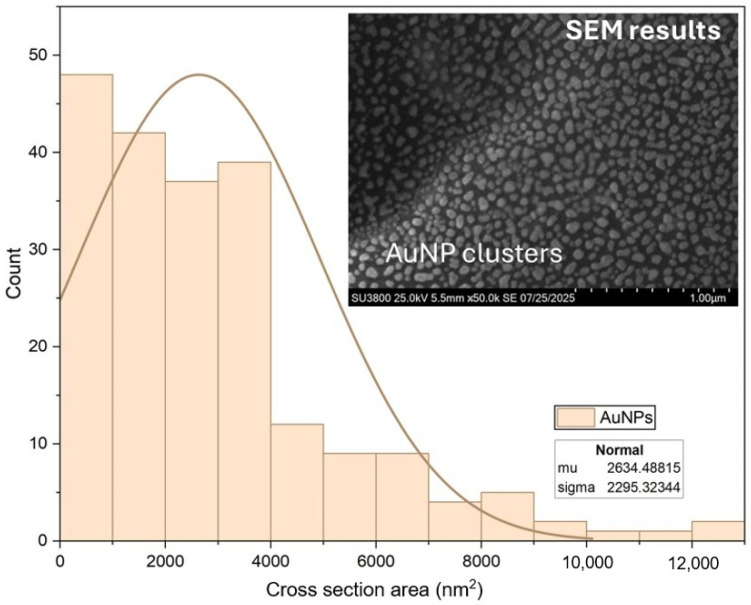
Size distribution of AuNP clusters. The histogram shows the frequency of AuNP cluster areas, with a normal distribution fit (inset table: mean = 2634 nm^2^, standard deviation = 2295 nm^2^); SEM image (inset) illustrates the clustered morphology of AuNPs (Scale bar = 1 µm).

SEM analysis showed AuNPs clusters sample that were not located directly on the cellulose fibers, while the AFM results of a difference sample confirmed that AuNPs reside on the fiber surfaces. The SEM-derived cluster sizes, analyzed using ImageJ software (version 1.54f, National Institutes of Health, USA), follow a normal distribution, with a mean area of ~2634 nm^2^ (Figure 7). These findings suggest that AuNPs tend to form clusters, but their spatial location depends on sample conditions, which may affect the material’s plasmonic properties and potential applications.

### 3.3. Functional Analysis and Net Charge Analysis Using FTIR and PCD

#### 3.3.1. FTIR Analysis of Cellulose Modified with APTES and AuNPs

**Figure 8:**
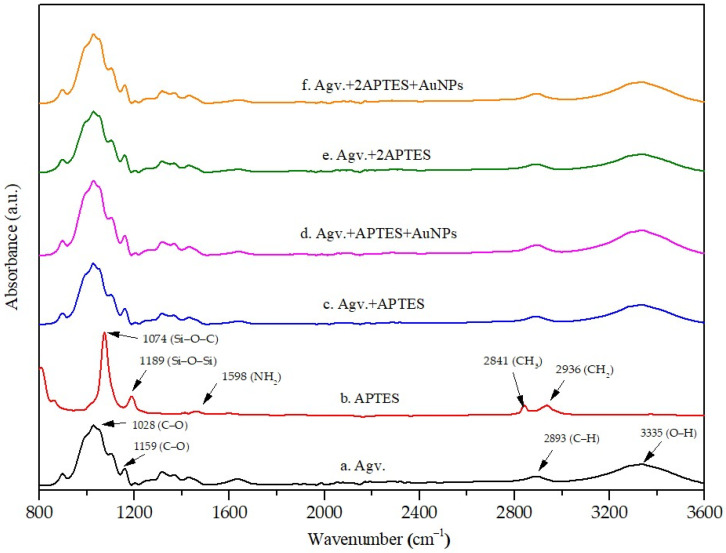
FT-IR spectra of a. Agv. Cellulose, b. 3-aminopropyltriethoxysilane (APTES), c. Agv. Cellulose treated with APTES, d. Agv. Cellulose treated with APTES and AuNPs, e. Agv. Cellulose treated with 2APTES and f. Agv. Cellulose treated with 2APTES and AuNPs.

From Figure 8, The FTIR spectrum of Agave-derived cellulose exhibited characteristic polysaccharide bands, including a broad O–H stretching vibration at 3335 cm^−1^, C–H stretching at 2893 cm^−1^, and a band at 1631 cm^−1^ assigned to bound water

Peaks at 1028 and 1159 cm^−1^ correspond to glycosidic C–O–C and C–O stretching vibrations, confirming the structural integrity of natural cellulose [35]. After APTES functionalization, notable spectral changes were observed. The O–H stretching band near 3330 cm^−1^ decreased in intensity and shifted, indicating hydrogen bonding with the silanol and amine groups of APTES.

New absorption bands near 1102 cm^−1^ were attributed to Si–O–C and Si–O–Si linkages, confirming condensation reactions between cellulose hydroxyl groups and silane moieties [36,37]. These features provide strong evidence of successful APTES grafting onto the cellulose surface. Upon the incorporation of additional modifications were observed. The amine N–H bending band at approximately 1636 cm^−1^ broadened and intensified, suggesting coordination between the amine groups and AuNPs. Meanwhile, the O–H stretching band around 3333 cm^−1^ further decreased, consistent with interactions between hydroxyl groups and AuNPs. The comparative FTIR spectra of cellulose, APTES, and their AuNPs-modified composites are illustrated in Figure 8, clearly showing the shifts in the O–H, C–O, and Si–O bands following functionalization. At higher APTES concentrations, a reduction in C–O stretching at 1028 cm^−1^ implied shielding of cellulose functional groups by the silane network and anchoring of AuNPs. Collectively, these results suggest a dual interaction mechanism: covalent bonding of APTES to cellulose via Si–O–C linkages, and non-covalent stabilization of AuNPs through O–H···Au and N–H···Au interactions, consistent with previously reported nanoparticle–biopolymer systems [10,38].

#### 3.3.2. Particle Charge Detection (PCD)

From Table 1, PCD titration showed that agave cellulose exhibited a highly negative starting potential (−245 mV) but a very low charge equivalent (−16 µeq·L^−1^). The indicates that although the surface is strongly anionic, only a limited number of ionizable groups are accessible. Modification with APTES shifted the potential toward less negative values (−85 mV) and increased the charge equivalent (−148 µeq·L^−1^), confirming the successful introduction of protonatable amine groups that both reduce surface negativity and provide additional titratable sites. In contrast, cellulose combined with AuNPs alone displayed a substantial increase in charge equivalent (−362 µeq·L^−1^) but retained a highly negative potential (−227 mV), suggesting that AuNPs contribute additional surface charges without effectively neutralizing the cellulose surface. For the combined APTES + AuNPs system, the starting potential was nearly neutral (−35 mV), while the charge equivalent (−141 µeq·L^−1^) remained comparable to that of APTES-only cellulose, reflecting a balance between amine neutralization and nanoparticle contribution. Interestingly, doubling the APTES concentration in the AuNP-modified sample further stabilized the potential near neutrality (−31 mV) but reduced the charge equivalent (−43 µeq·L^−1^), likely due to multilayer silane coverage that shields accessible groups. Overall, these results demonstrate that APTES reduces surface negativity and increases charge capacity, while AuNPs provide additional charged sites. Their combination allows for precise tuning of the cellulose surface electrostatics for potential biomolecular and cellular interactions.

**Figure 9:**
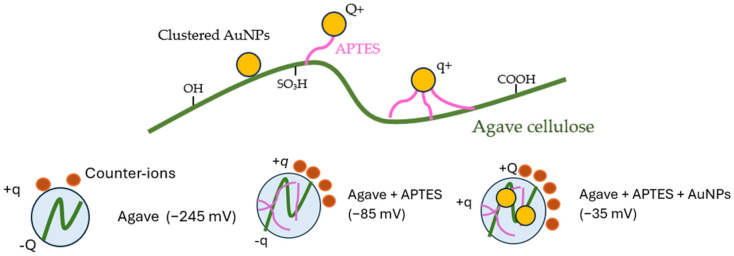
The diagram of counter-ion due to the charge of Agv. + APTES + AuNPs.

The effect of electrostatic interactions on AuNPs influences the charge distribution in composite materials composed of Agave cellulose, APTES and AuNPs. The pure Agave Cellulose composites exhibit a strong negative charge, whereas the Agv. + APTES + AuNPs composites show a reduced charge distribution (Figure 9). Additionally, this phenomenon is also related to the charge transfer among Agave cellulose, APTES and AuNPs.

## 4. Conclusions

The optical characterization of Agv.-based composites demonstrated the significant impact of AuNPs and surface functionalization with APTES on their absorption, fluorescence, and transmittance properties. Spectral analysis confirmed the presence of organic, plant-derived components, while the integration of AuNPs and APTES induced notable changes in optical behavior due to nanoparticle interactions and enhanced dispersion. Also, a distinct surface plasmon resonance (SPR) band of AuNPs was observed at around 530 nm. Notably, APTES played a crucial role in optimizing the spatial arrangement between the AuNPs and the plant-based matrix, enabling plasmon-enhanced cellulose auto-fluorescence. The enhancement of the autofluorescence signal increased about two times relative to the Agv. + AuNPs sample and 67% higher than the Agv. cellulose autofluorescence. These findings underscore the importance of controlled surface modification in fine-tuning the optical performance of Agv.-based nanocomposites. An optimal concentration of APTES was shown to enhance both fluorescence intensity and transmittance, making these materials promising candidates for applications in bioimaging, sensing, and optoelectronic devices.

## Figures and Tables

**Table 1:** Charge quantification of Agv. + APTES + AuNPs composite using PCD.

Sample	Equivalent (µeq/L)	Start Potential (mV)	Surface Interpretation
Agv.	−16	−245	Slightly negative surface charge due to deprotonated hydroxyl groups [39].
Agv. + APTES	−148	−85	Amino groups from APTES can be protonated (–NH_3_^+^), compensating for the negative charges; however, residual –OH and Si–O^−^ groups maintain the overall negative charge of the surface [40,41].
Agv. + AuNPs	−362	−227	Strongly negative surface charge resulting from citrate-stabilized AuNPs [4,42].
Agv. + APTES + AuNPs	−141	−35	APTES partially neutralizes the negative charges on citrate-coated AuNPs and facilitates their immobilization on cellulose, resulting in an almost neutral surface [43].
Agv. + 2APTES + AuNPs	−43	−31	A double-layer APTES coating increases the density of amino-silane groups, further compensating negative charges and approaching electrical neutrality [44].

## Data Availability

The original contributions presented in this study are included in the article/Supplementary Materials. Further inquiries can be directed to the corresponding author.

## Supplementary Materials

The following supporting information can be downloaded at: https://www.mdpi.com/article/10.3390/polym17233152/s1, Figure S1: Images of fluorescence microscopy: (A) Bright Filed image, (B) Fluorescence image of modified APTES with FITC on cellulose and (C) Fluorescence image of untreated APTES on cellulose; Figure S2: Absorption of AuNPs on cellulose compared with glass slide. Refs. [45,46,47,48,49] are cited in Supplementary Materials file.

## References

[1] Zhang Q., Zhu E., Li T., Zhang L., Wang Z. (2024). High-Value Utilization of Cellulose: Intriguing and Important Effects of Hydrogen Bonding Interactions—A Mini-Review. Biomacromolecules.

[2] Pinto R.J., Carlos L.D., Marques P.A., Silvestre A.J., Freire C.S. (2014). An overview of luminescent bio-based composites. J. Appl. Polym. Sci..

[3] Kulpinski P., Namyslak M., Grzyb T., Lis S. (2012). Luminescent cellulose fibers activated by Eu^3+^-doped nanoparticles. Cellulose.

[4] Ghosh S.K., Pal T. (2007). Interparticle coupling effect on the surface plasmon resonance of gold nanoparticles: From theory to applications. Chem. Rev..

[5] Petryayeva E., Krull U.J. (2011). Localized surface plasmon resonance: Nanostructures, bioassays and biosensing—A review. Anal. Chim. Acta.

[6] Kelly K.L., Coronado E., Zhao L.L., Schatz G.C. (2003). The optical properties of metal nanoparticles: The influence of size, shape, and dielectric environment. J. Phys. Chem. B.

[7] Jain P.K., Huang X., El-Sayed I.H., El-Sayed M.A. (2008). Noble metals on the nanoscale: Optical and photothermal properties and some applications in imaging, sensing, biology, and medicine. Acc. Chem. Res..

[8] Anker J.N., Hall W.P., Lyandres O., Shah N.C., Zhao J., Van Duyne R.P. (2008). Biosensing with plasmonic nanosensors. Nat. Mater..

[9] Turkevich J., Stevenson P.C., Hillier J. (1951). A study of the nucleation and growth processes in the synthesis of colloidal gold. Discuss. Faraday Soc..

[10] Iravani S. (2011). Green synthesis of metal nanoparticles using plants. Green Chem..

[11] Ahmed S., Ahmad M., Swami B.L., Ikram S. (2016). Green synthesis of silver nanoparticles using Azadirachta indica aqueous leaf extract. J. Radiat. Res. Appl. Sci..

[12] Wei H., Rodriguez K., Renneckar S., Vikesland P.J. (2014). Environmental science and engineering applications of nanocellulose-based nanocomposites. Environ. Sci. Nano.

[13] Musino D., Rivard C., Landrot G., Novales B., Rabilloud T., Capron I. (2021). Hydroxyl groups on cellulose nanocrystal surfaces form nucleation points for silver nanoparticles of varying shapes and sizes. J. Colloid Interface Sci..

[14] Geethalakshmi K., Ng T.Y., Crespo-Otero R. (2016). Tunable optical properties of OH-functionalised graphene quantum dots. J. Mater. Chem. C.

[15] Miao C., Mauran D., Hamad W.Y. (2022). How hydrogen-bonding interactions and nanocrystal aspect ratios influence the morphology and mechanical performance of polymer nanocomposites reinforced with cellulose nanocrystals. Soft Matter.

[16] Nagpal K., Rauwel E., Estephan E., Soares M.R., Rauwel P. (2022). Significance of hydroxyl groups on the optical properties of ZnO nanoparticles combined with CNT and PEDOT: PSS. Nanomaterials.

[17] Voicu S.I., Thakur V.K. (2021). Aminopropyltriethoxysilane as a linker for cellulose-based functional materials: New horizons and future challenges. Curr. Opin. Green Sustain. Chem..

[18] Amit S.K., Gomez-Maldonado D., Bish T., Peresin M.S., Davis V.A. (2024). Properties of APTES-Modified CNC Films. ACS Omega.

[19] Kochuveedu S.T., Kim D.H. (2014). Surface plasmon resonance mediated photoluminescence properties of nanostructured multicomponent fluorophore systems. Nanoscale.

[20] Khanjanzadeh H., Behrooz R., Bahramifar N., Gindl-Altmutter W., Bacher M., Edler M., Griesser T. (2018). Surface chemical functionalization of cellulose nanocrystals by 3-aminopropyltriethoxysilane. Int. J. Biol. Macromol..

[21] Vedhanayagam M., Nair B.U., Sreeram K.J. (2019). Effect of functionalized gold nanoparticle on collagen stabilization for tissue engineering application. Int. J. Biol. Macromol..

[22] Amendola V., Pilot R., Frasconi M., Maragò O.M., Iatì M.A. (2017). Surface plasmon resonance in gold nanoparticles: A review. J. Phys. Condens. Matter.

[23] Jahan M.S., Shamsuzzaman M., Rahman M.M., Moeiz S.I., Ni Y. (2012). Effect of pre-extraction on soda-anthraquinone (AQ) pulping of rice straw. Ind. Crops Prod..

[24] Seo J.-H., Kim H.-J. (2015). Effect of H_2_O_2_ bleaching with ultrasonication on the properties of thermomechanical pulp and unbleached kraft pulp. Ultrason. Sonochem..

[25] Fergusson J., Wallace G.Q., Sloan-Dennison S., Carland R., Shand N.C., Graham D., Faulds K. (2023). Plasmonic and photothermal properties of silica-capped gold nanoparticle aggregates. J. Phys. Chem. C.

[26] Kyaw H.H., Al-Harthi S.H., Sellai A., Dutta J. (2015). Self-organization of gold nanoparticles on silanated surfaces. Beilstein J. Nanotechnol..

[27] Lakowicz J.R. (2006). Principles of Fluorescence Spectroscopy.

[28] Hermanson G.T. (2013). Bioconjugate Techniques.

[29] Dulkeith E., Morteani A., Niedereichholz T., Klar T., Feldmann J., Levi S., Van Veggel F., Reinhoudt D., Möller M., Gittins D. (2002). Fluorescence Quenching of Dye Molecules near Gold Nanoparticles: Radiative and Nonradiative Effects. Phys. Rev. Lett..

[30] Persson B.N.J., Lang N.D. (1982). Electron-hole-pair quenching of excited states near a metal. Phys. Rev. B.

[31] Kim K.-S., Zakia M., Yoon J., Yoo S.I. (2019). Metal-enhanced fluorescence in polymer composite films with Au@Ag@SiO_2_ nanoparticles and InP@ZnS quantum dots. RSC Adv..

[32] Zhu Z., Yuan P., Li S., Garai M., Hong M., Xu Q.-H. (2018). Plasmon-enhanced fluorescence in coupled nanostructures and applications in DNA detection. ACS Appl. Bio Mater..

[33] Minopoli A., Della Ventura B., Campanile R., Tanner J.A., Offenhäusser A., Mayer D., Velotta R. (2021). Randomly positioned gold nanoparticles as fluorescence enhancers in apta-immunosensor for malaria test. Microchim. Acta.

[34] Anger P., Bharadwaj P., Novotny L. (2006). Enhancement and quenching of single-molecule fluorescence. Phys. Rev. Lett..

[35] Poletto M., Ornaghi H., Zattera A. (2014). Native cellulose: Structure, characterization and thermal properties. Materials.

[36] Socrates G. (2004). Infrared and Raman Characteristic Group Frequencies: Tables and Charts.

[37] Kawalerczyk J., Walkiewicz J., Dziurka D., Mirski R., Brózdowski J. (2022). APTES-modified nanocellulose as the formaldehyde scavenger for UF adhesive-bonded particleboard and strawboard. Polymers.

[38] Prodan D., Moldovan M., Furtos G., Saroși C., Filip M., Perhaița I., Carpa R., Popa M., Cuc S., Varvara S. (2021). Synthesis and characterization of some graphene oxide powders used as additives in hydraulic mortars. Appl. Sci..

[39] Habibi Y., Lucia L.A., Rojas O.J. (2010). Cellulose nanocrystals: Chemistry, self-assembly, and applications. Chem. Rev..

[40] Lu Q., Moore J.M., Huang G., Mount A.S., Rao A.M., Larcom L.L., Ke P.C. (2004). RNA polymer translocation with single-walled carbon nanotubes. Nano Lett..

[41] Wang J., Yang L., Xie J., Wang Y., Wang T.-J. (2020). Surface amination of silica nanoparticles using tris (hydroxymethyl) aminomethane. Ind. Eng. Chem. Res..

[42] Njoki P.N., Lim I.-I.S., Mott D., Park H.-Y., Khan B., Mishra S., Sujakumar R., Luo J., Zhong C.-J. (2007). Size correlation of optical and spectroscopic properties for gold nanoparticles. J. Phys. Chem. C.

[43] Pastoriza-Santos I., Liz-Marzán L.M. (2008). Colloidal silver nanoplates. State of the art and future challenges. J. Mater. Chem..

[44] Zhao J., Milanova M., Warmoeskerken M.M., Dutschk V. (2012). Surface modification of TiO2 nanoparticles with silane coupling agents. Colloids Surf. A Physicochem. Eng. Asp..

[45] Fan C., Wang S., Hong J.W., Bazan G.C., Plaxco K.W., Heeger A.J. (2003). Beyond superquenching: Hyper-efficient energy transfer from conjugated polymers to gold nanoparticles. Proc. Natl. Acad. Sci. USA.

[46] He Z., Li F., Zuo P., Tian H. (2023). Principles and applications of resonance energy transfer involving noble metallic nanoparticles. Materials.

[47] Campora L., Metzger C., Dähnhardt-Pfeiffer S., Drexel R., Meier F., Fürtauer S. (2022). Fluorescence Labeling of Cellulose Nanocrystals A Facile and Green Synthesis Route. Polymers.

[48] Nair R.R., Hyun J.H., Kim J., Jung K.O., Kim D. (2025). Recent progress in the development of cellulose-derived organic-nanopolymer and coordination network platforms for application as optical chemosensors. Adv. Compos. Hybrid Mater..

[49] Aziz T., Farid A., Haq F., Kiran M., Ullah A., Zhang K., Li C., Ghazanfar S., Sun H., Ullah R. (2022). A Review on the Modification of Cellulose and Its Applications. Polymers.

