# Supplementary material for: Impact of Hydrogen Bonding in Natural Cellulose Fibers on Plasmonic Nanoparticles
Source: Polymers (Basel). 2025 Nov 27;17(23):3152. doi: 10.3390/polym17233152 (PMC12694114; doi:10.3390/polym17233152)
Supplement: Supplementary file 1 [file polymers-17-03152-s001.zip › polymers-3878034-supplementary.pdf]

Support information

1. Fluorescence image of cellulose using APTES-FITC labelling

The autofluorescence spectroscopy results (Figure 4) revealed distinct emission behaviors of the Agv.-based composites. The native Agv. sample showed moderate autofluorescence intensity, with a peak between 400 and 460 nm, attributed to intrinsic plant-derived compounds. The addition of AuNPs resulted in decreased autofluorescence intensity, consistent with quenching mechanisms such as Forster resonance energy transfer (FRET) and surface energy transfer (SET) [1,2]. In contrast, the Agv. + APTES + AuNPs composite exhibited a pronounced autofluorescence enhancement, suggesting that APTES improved nanoparticle dispersion and stabilized interfacial interactions through hydrogen bonding with cellulose hydroxyl groups [3,4]. Such enhancement aligns with the known role of silane coupling agents in modulating the optical properties of hybrid nanomaterials [4].

A parallel trend was observed in fluorescence staining experiments. APTES-modified Agv. cellulose fibers exhibited strong fluorescence after FITC labeling (Figure S1, (B)), while unmodified cellulose showed no emission when stained with TRITC (Figure S1, (C)). These results can be explained by the chemical mechanism of dye conjugation. Native cellulose contains abundant hydroxyl groups but lacks primary amine groups, which are necessary for covalent bonding with isothiocyanate-based fluorophores such as FITC and TRITC. This contrast demonstrates that APTES introduces reactive amine groups essential for covalent fluorophore conjugation through thiourea linkages [5]. APTES's ability to facilitate both enhanced fluorescence spectroscopy responses and effective microscopic staining confirms its dual role in improving chemical reactivity and optical performance. These findings align with previous studies on silane-based surface modifications that enhance fluorescence and labeling efficiency in cellulose–nanoparticle systems [6,7].

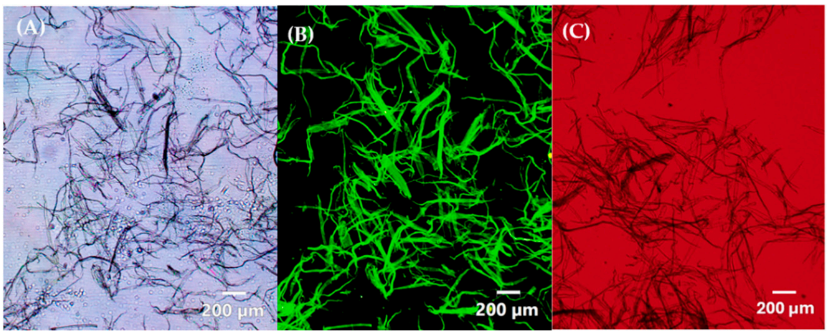

Figure S1. Images of fluorescence microscopy: (A) Bright Filed image, (B) Fluorescence image of modified APTES with FITC on cellulose and (C) Fluorescence image of untreated APTES on cellulose

2. FDTD simulation

The electromagnetic wave was simulated using the finite-difference time-domain (FDTD) method with ANSYS Lumerical software to analyze the absorption spectrum of AuNPs, as shown in Figure S2. The AuNPs were positioned on cellulose and glass slides. This observation revealed the effect of dipole-dipole

interactions between the AuNPs and the substrates. This result exhibited the same trend as the absorption data presented in Figure 3.

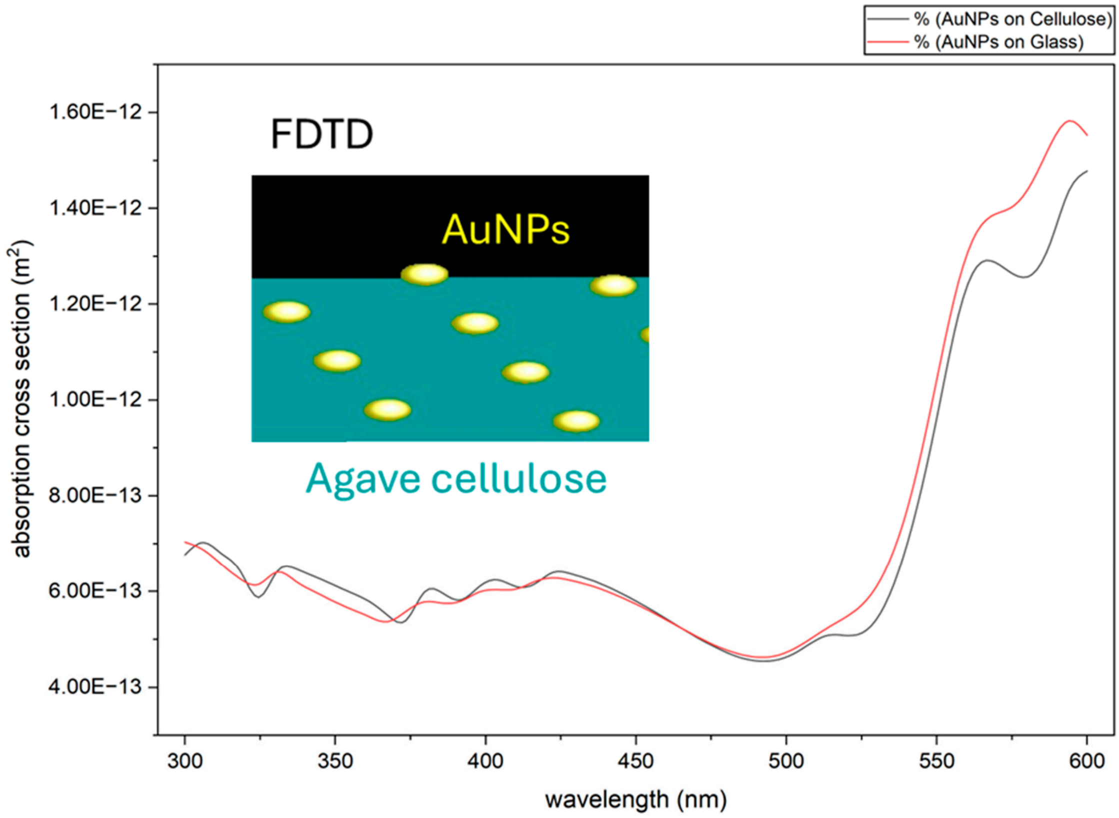

Figure S2. Absorption cross sections of AuNPs on cellulose compared with glass slide.

References

1. Fan, C.; Wang, S.; Hong, J.W.; Bazan, G.C.; Plaxco, K.W.; Heeger, A.J. Beyond superquenching: hyper-efficient energy transfer from conjugated polymers to gold nanoparticles. *Proceedings of the National Academy of Sciences of the United States of America* **2003**, *100*, 6297-6301, doi:10.1073/pnas.1132025100.
2. He, Z.; Li, F.; Zuo, P.; Tian, H. Principles and applications of resonance energy transfer involving noble metallic nanoparticles. *Materials* **2023**, *16*, 3083.
3. Hermanson, G.T. *Bioconjugate techniques*; Academic press: 2013.
4. Khanjanzadeh, H.; Behrooz, R.; Bahramifar, N.; Gindl-Altmutter, W.; Bacher, M.; Edler, M.; Griesser, T. Surface chemical functionalization of cellulose nanocrystals by 3-aminopropyltriethoxysilane. *International journal of biological macromolecules* **2018**, *106*, 1288-1296.
5. Campora, L.; Metzger, C.; Dähnhardt-Pfeiffer, S.; Drexel, R.; Meier, F.; Fürtauer, S. Fluorescence Labeling of Cellulose Nanocrystals A Facile and Green Synthesis Route. Polymers 2022, 14, 1820. **2022**.
6. Nair, R.R.; Hyun, J.H.; Kim, J.; Jung, K.O.; Kim, D. Recent progress in the development of cellulose-derived organic-nanopolymer and coordination network platforms for application as optical chemosensors. *Advanced Composites and Hybrid Materials* **2025**, *8*, 30.
7. Aziz, T.; Farid, A.; Haq, F.; Kiran, M.; Ullah, A.; Zhang, K.; Li, C.; Ghazanfar, S.; Sun, H.; Ullah, R.; et al. A Review on the Modification of Cellulose and Its Applications. *Polymers (Basel)* **2022**, *14*, doi:10.3390/polym14153206.
